# Anticipating visceral leishmaniasis epidemics due to the conflict in Northern Ethiopia

**DOI:** 10.1371/journal.pntd.0011188

**Published:** 2023-03-23

**Authors:** Carl Boodman, Johan van Griensven, Nitin Gupta, Ermias Diro, Koert Ritmeijer

**Affiliations:** 1 Clinical Investigator Program, University of Manitoba, Winnipeg, Canada; 2 Unit of HIV and Neglected Tropical Diseases, Institute of Tropical Medicine, Antwerp, Belgium; 3 Department of Infectious Diseases, Kasturba Medical College, Manipal, India; 4 Department of Internal Medicine, University of Washington, Seattle, United States of America; 5 Médecins Sans Frontières, Amsterdam, the Netherlands; Academic Medical Center: Amsterdam UMC Locatie AMC, NETHERLANDS

On November 4, 2020, conflict erupted between the Ethiopian federal government and the Northern Ethiopian regional forces called the Tigray People’s Liberation Front [1,2]. The crisis has since driven over 4 million people to flee their homes, often with little possessions and no shelter, remaining internally displaced in the Tigray, Afar, and Amhara regions known to be endemic for visceral leishmaniasis (VL) [1–4]. The Internal Displacement Monitoring Centre estimates that this conflict triggered the “highest displacement figures ever recorded for any country in any given year” [2]. An additional 59,000 people have sought safety in eastern Sudan, concentrated around Gedaref and southern Kassala states, also known to be highly endemic for VL [4,5]. East Africa currently composes the global epicenter of VL, accounting for 66% of the reported cases worldwide [6]. It also comprises the area with the highest rate of VL-HIV coinfection, further amplifying VL transmission [4,7]. Considering that conflict has previously triggered large VL epidemics with elevated case fatality, additional funding and operational coordination for a regional approach to VL screening, treatment and prevention will be required for the WHO to reach its goal of eliminating this neglected tropical disease as a public health problem by 2030 [4,5,8,9].

Visceral leishmaniasis is a vector-borne disease transmitted by sandflies [3,4]. This systemic disease affects the reticuloendothelial system and is fatal if untreated [4]. In East Africa, visceral leishmaniasis is caused by *Leishmania donovani* and is predominantly transmitted by the *Phlebotomus orientalis* sandflies that thrive in the *Acacia*-*Balanites* forests along Ethiopia’s north-western border with Sudan [3]. *Phlebotomus orientalis* is exophagic, preferring to bite the human host outside the home [10]. Thus, sleeping outdoors is a significant risk factor for VL, a factor that renders refugees without shelter particularly vulnerable to VL acquisition [9,10]. Since VL in East Africa is principally understood to be anthroponotic, human migration acts as one of the primary drivers of disease transmission [4,9].

Previous refugee crises have produced large deadly epidemics of VL in East Africa [5,8]. Within a year of conflict breaking out in South Sudan in 2013, cases of VL more than tripled in the Jonglei and Upper Nile States [5]. Earlier wars catalyzed devastating VL epidemics. After war exploded in South Sudan in 1983, approximately one third of the Western Upper Nile’s population of 280,000 died of VL during a 10-year period [8]. While conflict has long been known to trigger outbreaks of neglected tropical diseases, epidemics of VL are deemed to be the deadliest as conflict interferes with the provision of care for a fatal disease [5]. Furthermore, human migration introduces VL to new locations outside those of known endemicity. This phenomenon is well documented among Ethiopian migrant laborers who descend from high-altitude Amhara areas to work on commercial farms on the Sudanese border [3]. The massive forced displacement due to the war in Northern Ethiopia may cause VL outbreaks as well as VL introduction to new geographic foci.

The risk of VL epidemics due to the war in Northern Ethiopia is further intensified by widespread immunosuppression in the forms of HIV and malnutrition. Individuals coinfected with VL and HIV act as super-spreaders for VL as their diminished cellular immunity allows *Leishmania* amastigotes to proliferate [4,7]. Since 2020, the war in Northern Ethiopia has been associated with innumerable accounts of sexual violence [11]. Considering that Ethiopia currently exhibits the highest rates of VL-HIV coinfection, with approximately 20% to 30% of VL patients coinfected with HIV, large-scale rape will increase HIV transmission, a phenomenon which may indirectly act to amplify VL transmission [7]. Malnutrition also diminishes cellular immunity and is a known risk factor for VL [4,5]. At the time of writing, it is estimated that 9 million people across Ethiopia’s Northern regions require food aid to avoid malnutrition and almost 40% of the Tigrayan population experience an extreme lack of access to food [11]. Malnutrition on such a massive scale is likely to amplify VL transmission.

A framework for a coordinated approach to eliminating VL in East Africa has been proposed recently, involving enhanced disease surveillance, increased availability of therapeutics, operational research, and vector control [4]. Such a coordinated effort is needed now more than ever. As the massive displacements triggered by the war in Northern Ethiopia overlay precisely with areas with the highest VL transmission in the world, budgetary allocations for VL elimination programs calculated before this conflict will underestimate the required resources. Furthermore, an operational strategy is needed to maintain and rebuild essential health services during and after conflict. The global health community should anticipate VL epidemics as a consequence of the conflict in Northern Ethiopia. Incorporating the effect of this war on VL dynamics is necessary for the WHO to achieve its 2030 elimination target.
